# Tip up - Simplified technique for non-surgical rhinoplasty: A case series

**DOI:** 10.1016/j.jobcr.2025.01.013

**Published:** 2025-02-12

**Authors:** Cláudia Almeida, Victor Rogerio, Gabriela Giro, Victor Munoz-Lora, Marcelo Germani

**Affiliations:** a Private Office, Recife, Brazil; b Private Office, São Paulo, Brazil; c Department of Periodontology and Implantology, University of Guarulhos, São Paulo, Brazil; d Let's HOF Academy, São Paulo, Brazil

**Keywords:** Hyaluronic acid, Nose, Fillers

## Abstract

Non-surgical nasal rhinoplasty using hyaluronic acid (HA) is a technique that has been widely adopted due to its minimally invasive and reversible nature. Reshaping the nose with HA is becoming increasingly popular for its quick and safe outcomes, serving as an effective alternative for correcting minor nasal deformities. The proposed approach, named the Tip Up (TU) technique, focuses on strategic injection sites, specifically in the Nasal Tip region, Nasal Spine, and Columella, utilizing minimal amounts of the product to minimize risks and enhance the predictability of the results. Procedures are performed under anesthesia without vasoconstrictors to prevent ischemia, and careful selection of the product ensures subtle and precise corrections. This case series details all aspects of the TU technique, highlighting the satisfactory and predictable aesthetic results achieved through a safe and reliable procedure.

## Graphical abstract

The Tip Up (TU) technique utilizes strategic injection sites for non-surgical nasal rhinoplasty with hyaluronic acid, ensuring safe and predictable aesthetic results.

**Figure:**
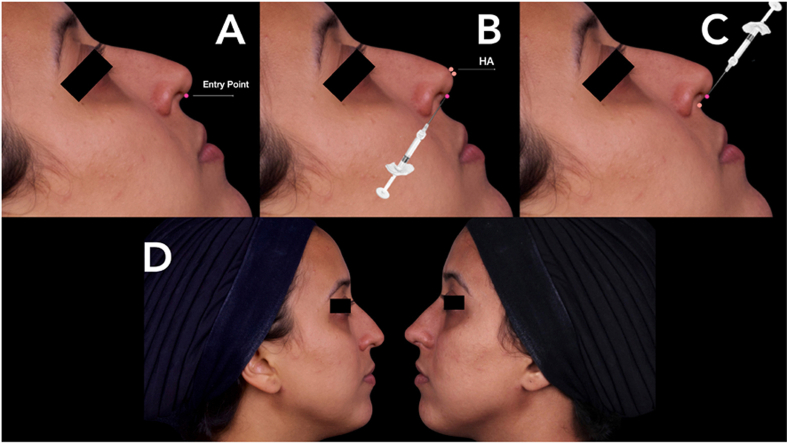

## 1 Introduction

Non-surgical nasal rhinoplasty using Hyaluronic Acid (HA) is gaining popularity as a minimally invasive, reversible technique with fewer risks of complications. Due to its rapid results and safety, it is considered a viable alternative for patients seeking to correct minor changes and/or nasal deformities.^1^^,^^2^ Anatomical knowledge is crucial to ensure predictable and safe outcomes. The choice between devices, whether a cannula or needle, for filler injection can significantly impact the safety of the procedure. While some studies suggest that needles may be preferable due to their ability to precisely select the application plane, especially in the deeper planes of the nasal dorsum where vasculature is more superficial, this type of device may increase the risk of tissue injuries such as hematomas and edema.^2^^,^^3^ The safety of the procedure is not guaranteed solely by anatomical knowledge and device choice; other factors, such as the reversibility of hyaluronic acid, the administration of small doses, and a slow, delicate application, are also essential to minimize the risk of adverse events.^4^ In clinical practice, professionals often adopt a holistic approach to the nose, starting at the anterior nasal spine, progressing through the columella and nasal tip, and culminating in the nasal dorsum. The latter region presents particularly complex vascularization, with fine branches from the internal carotid artery, such as the dorsal nasal artery, a branch of the ophthalmic artery. Due to this anatomical configuration and specific anastomoses in the dorsal region of the nose, there is a potential risk of amaurosis.^5^

To address these concerns, we propose a non-surgical nasal rhinoplasty approach using the simplified Tip Up (TU) technique. This approach employs a cannula exclusively, using a smaller amount of product and performing interventions solely in the Nasal Tip, Nasal Spine areas, and collumela thereby enhancing the safety and predictability of the procedures.

## 2 Case series

### 2.1 Application technique

All patients underwent a thorough anamnesis and clinical evaluation before treatment. Prior to the procedure, patients' faces were cleansed with 2 % alcoholic chlorhexidine. Local anesthesia was then administered in the Tip and Nasal Spine areas, using a total of 0.1 ml of 2 % lidocaine without vasoconstrictor (Xylestesin 2 % without vasoconstrictor – Cristália, Itapira, São Paulo, Brazil). The injection site chosen is precisely where the puncture will be made. It is advisable to avoid anesthetics containing vasoconstrictors to minimize the confounding effects caused by ischemia induced by the vasoconstrictor.

**Fig. 1:**
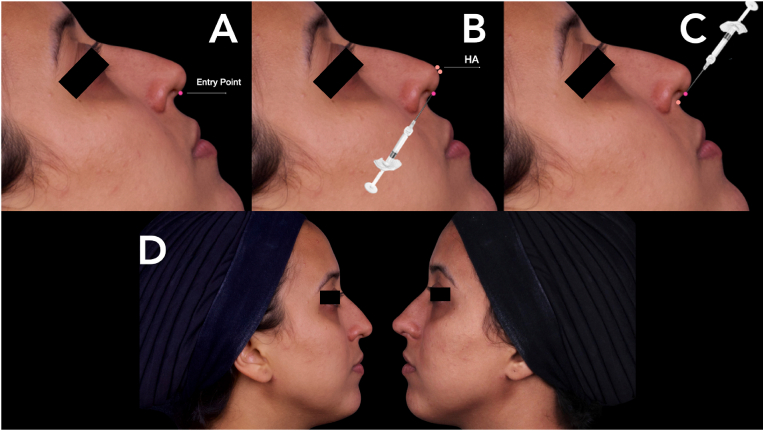
A, B, C: Tip Up Injection technique; D: Before and after Tip Up Technique.

After anesthesia, the puncture is made in the middle region of the columella using a needle (20G or 22G). A 22-gauge cannula (Prodeep, Allur Medical, Novo Hamburgo, Porto Alegre, Brazil) is inserted into the puncture with the opening facing the tissue, and directed towards the nasal tip, where small bolus are injected in this area. The cannula is then removed and reinserted, but now towards the nasal spine, touching the bone with the cannula's opening facing the bone margin, and small bolus are injected in this area, followed by a retroinjection in the columella region with the cannula's opening facing the tissue, and the cannula is removed (Fig. 1A, B and C).

In Clinical Cases 1 to 4, 0.4 ml of hyaluronic acid was injected in the Nasal Tip area (2 bolus of 0.1 ml each), the anterior nasal spine (0.1 ml bolus), and the columella (0.1 ml retro-injection) using different products: Belotero Intense (hialuronic acid concentration 25,5 mg/ml) in Case 1, Restylane Lyft (hialuronic acid concentration 20 mg/ml) in Cases 2 and 3, and Juvederm Voluma (hialuronic acid concentration 20 mg/ml) in Case 4. In Case 5, a total of 0.5 ml of Belotero Intense was injected in the same areas, with 3 bolus of 0.1 ml each in the Nasal Tip. A 22G cannula was used in all procedures.

**Fig. 2:**
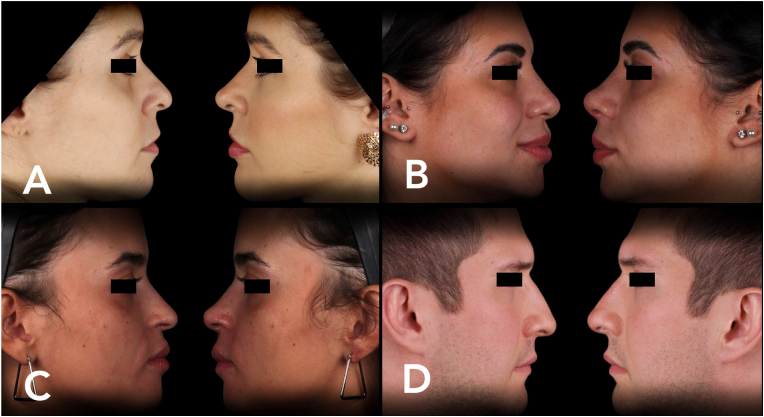
A, B, C, and D - The photos illustrate the results obtained 45 days after the procedure.

In all injection areas, the amount of product is individualized for each patient (Table 1). No prior accommodation or massage is recommended after the procedure. The results of the cases presented are shown in Fig. 1, Fig. 2A, B, C, and D. The photos illustrate the results 45 days after the procedure.

**Table 1:** Relevant characteristics/properties of a gel for use in the nose.

Properties	Expressiveness
**Elastic modulus**	High
**Tissue projection capacity**	High
**Cohesiveness**	Medium/High
**Reversibility**	High

As a criterion for inclusion in this patient case series, it is noteworthy that the absence of irregularities in the nasal dorsum region is a defining factor. This criterion excludes patients who require direct intervention in the nasal dorsum to achieve significant clinical outcomes.

In the simplified TU technique, the approach is focused exclusively on the areas of the nasal spine, nasal tip (Tip Nasal), and columella, eliminating the need to inject product into the dorsum and thereby reducing the total amount of product injected, making the procedure more predictable and safer.

The correct selection of the product for the technique is essential for the success of the clinical procedure. For interventions in the nasal region, it is advisable to choose a hyaluronic acid-based gel that exhibits high firmness, consequently a high elastic modulus. Other relevant characteristics are detailed in Table 2^6^.

**Table 2:** Products description, quantity per area and total used in each clinical case.

Injection Area	Case 1 (Fig. 1D) Belotero Intense	Case 2 (Fig. 2A) Restylane Lyft	Case 3 (Fig. 2B) Restylane Lyft	Case 4 (Fig. 2C) Juvederm Voluma	Case 5 (Fig. 2D) Belotero Intense
**Nasal Tip (Bolus)**	2 Bolus of 0.1 ml (each)	2 Bolus of 0.1 ml (each)	2 Bolus of 0.1 ml (each)	2 Bolus of 0.1 ml (each)	3 Bolus of 0.1 ml (each)
**Nasal Spine (Bolus)**	0.1 ml	0.1 ml	0.1 ml	0.1 ml	0.1 ml
**Columella (Retroinjection)**	0.1 ml	0.1 ml	0.1 ml	0.1 ml	0.1 ml
**Total amount of product**	0.4 ml	0.4 ml	0.4 ml	0.4 ml	0.5 ml

## 3 Discussion

Due to its predictable results and safety, the TU technique can be considered a viable alternative for both experienced and less experienced practitioners. By targeting strategic areas and avoiding regions with higher risk potential, and by using a safe device such as a cannula, this technique minimizes the chances of complications and maximizes the efficacy of the results.^4^^,^^7^ Given the complex vascular anatomy, the nasal region has a high incidence of adverse events, particularly vascular complications. In conventional rhinoplasty, the procedure involves a comprehensive approach to the nose, beginning from the anterior nasal spine, progressing through the columella and nasal tip, and extending to the nasal dorsum. The nasal dorsum is an area with intricate vascularization, traversed by delicate branches of major arteries, such as the dorsal nasal artery, a branch of the ophthalmic artery. Consequently, the risk of vascular complications is elevated when addressing the nasal dorsum.^3^^,^^11^

Additionally, the TU technique uses a smaller amount of product, enhancing predictability. The technique also allows for a quicker and less painful recovery for the patient, making it extremely suitable for those seeking aesthetic improvement with minimal invasion.^8^

Another highly relevant point is that different brands of HA-based products were used in the cases presented, all of which share a common feature: firmness. This feature is considered crucial in selecting the product for the nasal region. Some authors argue that highly cohesive products should be the primary choice due to their resistance to disintegration, which allows for a more effective permanence in the implanted region.^6^^,^^9^^,^^10^ Conversely, other authors suggest that products with low cohesiveness might be more suitable because they have less migration, thus ensuring easier permanence at the implant site. As observed, the presented cases were successful using firm products with varying levels of cohesiveness, further reinforcing the importance of firmness when choosing a gel for the nasal region.^4^^,^^10^

The reversibility provided by the degradation of HA by the enzyme hyaluronidase is another consideration when choosing a gel for procedures in the nasal area. All HA-based gels used in the respective clinical cases have extensive literature related to the subject; all are sensitive to the enzyme and show fast degradation when exposed to hyaluronidase.^10^^,^^11^

An aspect to consider in this series of cases is the importance of careful patient selection. Individuals with irregularities in the nasal dorsum may not experience the same benefits from this approach. However, it is crucial to highlight that, in many cases, improvement in the nasal tip alone already provides a significant advancement in the overall aesthetic aspect of the nose, as structuring this area enhances the continuity of the nasal dorsum.

The Tip Up technique ensures safety and predictability in non-surgical nasal rhinoplasty procedures by utilizing a minimal amount of product and avoiding high-risk anatomical areas, such as the nasal dorsum, where vascular structures are more concentrated. Furthermore, the use of a cannula in TU technique as described: a 22-gauge cannula is inserted and directed towards the nasal tip and small bolus are injected in this area, after that the cannula is then removed and reinserted, but now towards the nasal spine and small bolus are injected in this area, followed by a retroinjection in the columella region; reduces the risk of vascular obstruction. The use of a minimal amount of product delivered with cannula, targeted to specific areas of the nose as outlined by the TU technique, introduces distinct advantages and yields satisfactory outcomes, as demonstrated in this series of cases.

## 4 Conclusion

Based on the detailed results of the clinical case series, the TU technique can be considered a predictable, safe, and minimally invasive alternative for non-surgical nasal rhinoplasty, offering aesthetically significant results with a minimal amount of product.

## Ethics approval

The article titled "Tip Up - Simplified Technique for Non-Surgical Nasal Rhinoplasty: A Case Series" was submitted to ethics approval on September 30, 2023. The proposing institution for this research is Universidade Guarulhos - UNG. The current status of the project version is approved and is with the responsible researcher. The primary sponsor of the research is self-funded, indicating that the study did not receive external financial support.

## Funding

The article titled "Tip Up - Simplified Technique for Non-Surgical Nasal Rhinoplasty: A Case Series" authored by Cláudia Almeida DDS., M.Sc., Victor Rogerio DDS., Gabriela Giro DDS., Ph.D., M.Sc., Victor R. M. Munoz-Lora DDS., Ph.D., and Marcelo Germani DDS., M.Sc., does not report any form of financial support or funding. The research and publication of this work were conducted independently by the authors.

## Declaration of competing interest

Marcelo Germani and Victor Rogerio are speakers for Galderma, while Claudia Almeida is a speaker for Merz. Gabriela Giro and Victor Lora does not have any conflicts of interest.
